# Pre-pregnancy overweight or obesity moderates the association between prenatal maternal depressive symptoms and infant cord blood omega-3 levels

**DOI:** 10.1186/s12884-024-06732-4

**Published:** 2024-08-14

**Authors:** Lauren A. Costello, Katherine Ziegler, Lacey McCormack, Anahid Akbaryan, Julianna Collazo Vargas, William S. Harris, Kristina H. Jackson, Maria Barber, Santiago Morales, Amy J. Elliott, Christine Hockett, Lauren C. Shuffrey

**Affiliations:** 1 Department of Child and Adolescent Psychiatry, NYU Grossman School of Medicine, One Park Avenue, 8th Floor, New York, NY 10016 USA; 2 Avera Research Institute, Sioux Falls, SD 57108 USA; 3 Department of Pediatrics, University of South Dakota School of Medicine, Sioux Falls, SD 57108 USA; 4 Department of Internal Medicine, University of South Dakota School of Medicine, Sioux Falls, SD 57108 USA; 5 Fatty Acid Research Institute, Sioux Falls, SD 57106 USA; 6 OmegaQuant Analytics, LLC, Sioux Falls, SD 57106 USA; 7 Department of Psychology, University of Southern California, Los Angeles, CA 90007 USA

**Keywords:** Perinatal depression, Maternal mental health, Maternal BMI, Infant cord blood, Omega-3

## Abstract

**Background:**

Empirical evidence has demonstrated associations between pre-pregnancy obesity and perinatal maternal depressive symptoms. Omega-3 is an essential fatty acid derived from dietary sources that is critical for fetal brain development. Pre-pregnancy obesity is associated with higher omega-3 intake, but a weaker association between dietary intake and respective maternal and cord blood omega-3 levels. Further, lower intake of omega-3 during pregnancy has been linked to higher depressive symptoms. Yet, prior studies have not examined the interactive effects of pre-pregnancy overweight or obesity (OWOB) and prenatal maternal mental health symptoms on infant cord blood omega-3 levels.

**Methods:**

Participants included 394 maternal-infant dyads from the NIH Environmental influences on Child Health Outcomes (ECHO) - Safe Passage Study in South Dakota. A pre-pregnancy body mass index (BMI) > 25 was used to dichotomize participants as OWOB (54%) vs. non-OWOB (46%). Prenatal maternal depressive symptoms were measured using the Edinburgh Postnatal Depression Scale (EPDS) and prenatal maternal anxiety symptoms were measured using the State-Trait Anxiety Inventory (STAI). We implemented linear regression models to examine the interaction term between pre-pregnancy BMI category and prenatal maternal mental health symptoms on cord blood omega-3 levels. Secondary analyses were stratified by pre-pregnancy BMI category.

**Results:**

We observed a significant interaction between pre-pregnancy BMI category and prenatal maternal depressive symptoms with cord blood omega-3 (F(4,379) = 6.21, *p* < .0001, adj. R^2^ = 0.05). Stratified models revealed an association between prenatal maternal depressive symptoms with lower cord blood omega-3 levels only among individuals with pre-pregnancy OWOB (β = -0.06, 95% CI = -0.11, -0.02; F (2,208) = 4.00, *p* < .05, adj R^2^ = 0.03). No associations were observed among non-OWOB participants.

**Conclusions:**

Findings suggest maternal-placental transfer of omega-3 may represent one pathway by which maternal metabolic and mental health impacts infant development.

## Introduction

Empirical evidence has demonstrated an association between pre-pregnancy overweight and obesity (OWOB) and increased perinatal maternal mood symptoms [1–4]. Pre-pregnancy OWOB affects approximately 24% of individuals in the United States (US) [5]. Studies have shown that up to 20% of women develop a perinatal mental health illness, such as depression or anxiety, either during pregnancy or within a year of giving birth [6]. Further, the prevalence of obesity and mental health concerns during pregnancy has been rapidly increasing over the past 20 years [7–9]. Each diagnosis is associated with an increased risk for neurodevelopmental conditions or behavioral problems in the offspring, such as increased early childhood behavioral problems [10–13] or increased risk for attention deficit hyperactivity disorder (ADHD) [12, 14] and autism spectrum disorder (ASD) [12, 14–16] mediated through independent or overlapping fetal programming mechanisms [17–23].

Omega-3, an essential fatty acid derived from dietary sources critical for healthy fetal brain development [24], has been linked to numerous child health and neurodevelopmental outcomes. For example, higher supply of long-chain polyunsaturated fatty acids (LC-PUFA) in pregnant persons has been found to reduce the risk of preterm birth [25, 26] and is associated with improved sustained attention, working memory, and inhibitory control in infants by 27 months [27–30]. During the third trimester, the fetal brain undergoes a rapid growth and requires an additional 50–70 mg/day of DHA, in addition to normal maternal needs [31]. Therefore, fetal plasma and brain omega-3 concentrations are highly impacted by maternal diet [32].

Several observational studies have examined dietary FA intake patterns among pregnant persons [31, 33–38]. A pilot study in Europe investigated the link between dietary fat intake during early pregnancy and the FA composition of maternal plasma and umbilical cord plasma phospholipids, revealing associations between higher intake of Linoleic acid, Eicosapentaenoic acid (EPA), and Docosahexaenoic acid (DHA) during pregnancy and their increased presence in maternal plasma phospholipids [33]. Subsequent research further confirmed a direct association between dietary FA intake and corresponding concentrations in maternal blood and cord plasma phospholipids, particularly for most PUFAs [36]. Emerging data suggests that pre-pregnancy obesity is associated with higher dietary intake of LC-PUFA during pregnancy [36, 39], but lower plasma concentrations of omega-3 PUFA [40, 41]. These findings align with previous evidence found in both human [42] and animal [43, 44] models that pre-pregnancy obesity may impact the metabolism of FAs during pregnancy with potential downstream effects on infant FA levels.

Emerging literature suggests deficiencies of omega-3 FAs, which result from inadequate intake or depletion during pregnancy, may be implicated in the risk for perinatal mental health symptoms [45–48].

Cross-sectional analyses have demonstrated that lower concentrations of omega-3 fats in breastmilk (mainly DHA), as well as lower natural rates of seafood consumption, are robustly correlated with high rates of postnatal depression [49]. Other research has suggested that supplementation of EPA-rich oil may reduce some depressive symptoms during pregnancy and after childbirth [50]. Similarly, there is a relationship between the intake of omega-3 FAs and prenatal anxiety in both animal and human models [51–54]. In humans, Vaz et al. (2013) found that a lower intake of omega-3 FAs and a lower frequency of fish and seafood consumption were associated with a higher incidence of anxiety symptoms in pregnant women [52]. Further, a recent longitudinal study found that lower plasma omega-3 fatty acids and higher omega-6:omega-3 fatty acid ratios were associated with higher antenatal anxiety, but not postpartum anxiety [55].

Despite the wealth of literature on the effects of prenatal obesity or mental health symptoms on adverse offspring health outcomes, prior studies have not examined the interactive effects of pre-pregnancy overweight or obesity (OWOB) and prenatal mental health symptoms on infant cord blood omega-3 levels. In fact, research to date has exclusively focused on associations between *maternal* omega-3 intake or levels and *child* neurodevelopmental outcomes [56–58]. Thus, our objective was to determine the joint and independent associations of pre-pregnancy OWOB and prenatal mental health symptoms on infant cord blood omega-3 levels. Given the rising interest in the co-occurrence of these conditions [59, 60], there is important public health and clinical value to understanding the interactive effects of maternal metabolic and mental health on infant cord blood omega-3 levels.

## Methods

### Study design and participants

Participants were a subset of mother-infant dyads enrolled in the NIH funded Safe Passage Study in Rapid City or Sioux Falls, South Dakota sites of the Prenatal Alcohol and SIDS and Stillbirth (PASS) Network (see Dukes et al., 2014 for full study description) [61]. Eligibility criteria for the NIH Safe Passage Study comprised the capability to offer informed consent in English, being aged 16 or above at the time of consent, and a gestational age ranging from 6 weeks to 40 weeks upon consent, as determined by the estimated delivery date [61]. Inclusion criteria for the current study consisted of prior enrollment in the Safe Passage Study clinics in Rapid City or Sioux Falls, South Dakota, available prenatal maternal mental health data, available pre-pregnancy weight data derived from medical record abstractions, and available infant omega-3 data (*N* = 384). No participants were excluded from this analysis. Institutional review board (IRB) approvals were obtained for all PASS entities (clinical sites and laboratory centers) from the Health Research Ethics Committee of Avera Research Institute.

### Pre-pregnancy overweight or obesity

Pre-pregnancy BMI was measured via self-reported pre-pregnancy weight and clinical coordinator measured height, both captured at the recruitment interview. Maternal-infant medical charts were abstracted to obtain maternal and infant health information including maternal age at delivery, infant gestational age at birth, and the infant’s assigned biological sex at birth.

#### Prenatal maternal depressive symptoms

Information regarding maternal mental health during pregnancy was obtained at 20–24 weeks’ gestation. Depressive symptoms were assessed using the Edinburgh Postnatal Depression Scale (EPDS), a screening tool tailored to gauge symptoms of depression in perinatal women, with elevated scores correlating with increased severity of depressive symptoms [62]. Based on prior work, we used a cut-off score of ≥ 11 to indicate elevated maternal depressive symptoms [63].

### Prenatal maternal anxiety symptoms

Maternal anxiety symptoms were assessed using the State-Trait Anxiety Inventory (STAI), an anxiety screening tool designed to differentiate anxiety symptoms from depressive symptoms [64]. The STAI incorporates two subscales: state anxiety, indicating the current anxiety level of the participant during questionnaire completion, and trait anxiety, which is believed to remain stable over time and reflect inherent personality traits [65, 66]. Based on prior work, we used a cut- off score of > 40 on the STAI- state subscale to indicate state anxiety and a cut- off score of > 40 on the STAI- trait subscale to indicate trait anxiety [67].

### Neonatal cord blood collection

Cord blood was collected after delivery by research nurses. The cord was clamped at both ends and cut in between the clamps. After the infant was handed off to a medical provider or the mother, the research team removed the stopper tube, unclamped the cord and allowed blood to flow from the cord cut end into a K2 EDTA blood collection tube, and replaced the stopper which resulted in collecting a mixture of arterial and venous blood. Next, the sample was labeled and sent to the laboratory for storage and processing. Specimens were labeled and stored in a 4 °C refrigerator in the delivery ward within 60 min of collection. Samples were then aliquoted from the K2 EDTA tubes into cryovials and were stored at -80 °C until processing.

### Infant cord blood omega-3 assays

Cord blood samples were received at OmegaQuant and blood was spotted on filter paper that was pre-treated with a cocktail solution (Fatty Acid Preservative Solution, FAPS™) and allowed to dry at room temperature for 15 min. One punch of the dried blood spots was transferred to a screw-cap glass vial followed by addition of BTM (methanol containing 14% boron trifluoride, toluene, methanol; 35:30:35 v/v/v) (Sigma-Aldrich, St. Louis, MO). The vial was briefly vortexed and heated in a hot bath at 100˚C for 45 min. After cooling, hexane (EMD Chemicals, USA) and high-performance liquid chromatography (HPLC) grade water was added, the tubes were recapped, vortexed and centrifuged to separate layers. An aliquot of the hexane layer was transferred to a GC vial. GC was carried out using a GC-2010 Gas Chromatograph (Shimadzu Corporation, Columbia, MD) equipped with a SP-2560, 100-m fused silica capillary column (0.25 mm internal diameter, 0.2 μm film thickness; Supelco, Bellefonte, PA).

Fatty acids were identified by comparison with a standard mixture of fatty acids characteristic of red blood cells (RBC) (GLC OQ-A, NuCheck Prep, Elysian, MN) which was also used to construct individual fatty acid calibration curves. The following 24 fatty acids (by class) were identified: saturated (14:0, 16:0, 18:0, 20:0, 22:0 24:0); *cis* monounsaturated (16:1, 18:1, 20:1, 24:1); trans (16:1, 18:1, 18:2); *cis* n-6 polyunsaturated (18:2, 18:3, 20:2, 20:3, 20:4, 22:4, 22:5); *cis* n-3 polyunsaturated (18:3, 20:5, 22:5, 22:6). Fatty acid composition was expressed as a percent of total identified fatty acids. The omega-3 index is defined as the sum of 20:5n-3 (EPA) and 22:6n-3 (DHA) adjusted by a regression equation (*r* = .97) to predict the omega-3 index in the RBC.

### Statistical analyses

Data were analyzed using R version 4.1.3. Descriptive statistics were used to describe the characteristics of our study population. We dichotomized participants as not overweight or obese prior to pregnancy (non-OWOB) based on a pre-pregnancy BMI of < 25 or as overweight or obese prior to pregnancy (OWOB) based on a pre-pregnancy BMI of *≥* 25 [68]. Next, we conducted a series of three unadjusted linear regression models to examine the interaction term or main effects of pre-pregnancy OWOB (BMI *≥* 25) and (1) prenatal maternal depressive symptoms, (2) prenatal maternal state anxiety symptoms, and (3) prenatal maternal trait anxiety symptoms on the infant cord blood omega index. The final models were adjusted for preterm birth and infant assigned sex at birth. For all models, we report the regression coefficients (β) and the standard error of estimates for each main effect and the significant interaction term.

## Results

### Sample characteristics

**Fig. 1:**
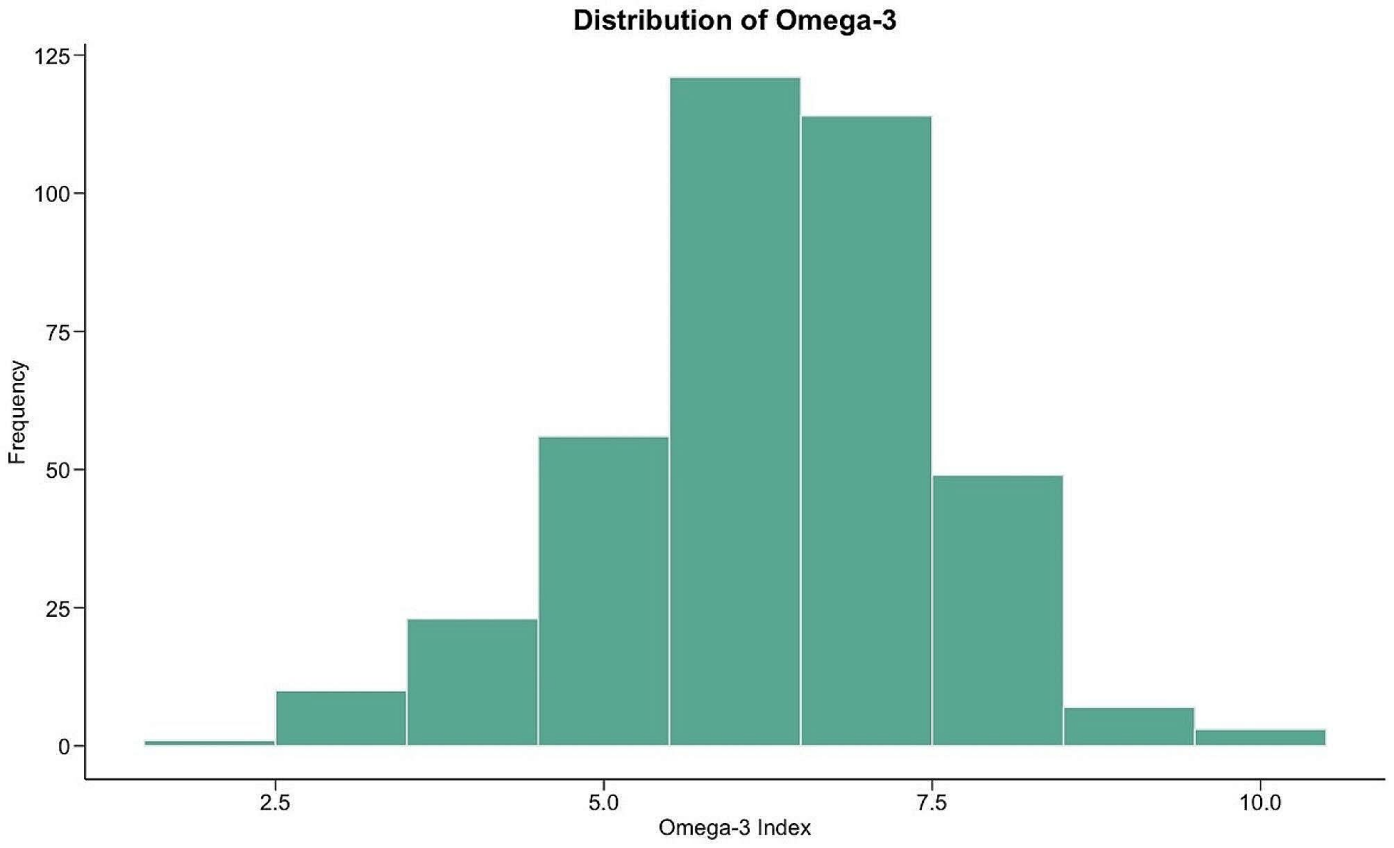
Distribution of infant Omega-3 levels at birth

Our final sample comprised of 384 mother-infant dyads (Table 1). Infants were born between 2010 and 2015. Pregnant persons primarily self-identified as White (85%) followed by American Indian or Alaskan Native (13%) and as Non-Hispanic or Latino (95%). Our sample had high levels of educational attainment with 35% of pregnant persons reporting completing college and 25% reporting completing High School. The majority of pregnant persons were Married or Partnered (93%). Median monthly household income per person was $1,160, not adjusted for inflation. The majority of pregnant persons had commercial health insurance or commercial health maintenance organization (HMO) (68%) followed by public assistance (31%). A total of 55% of participants were categorized as overweight or obese prior to pregnancy (OWOB) (*N* = 211) and a total of 45% of participants were categorized as not overweight or obese prior to pregnancy (non-OWOB) (*N* = 173). Pregnant participants in this sample had an average depressive symptom score on the EPDS of 4.70 ± 3.83, an average state anxiety score on the STAI of 26.0 ± 6.88, and an average trait anxiety score on the STAI of 30.7 ± 8.15. Using well-established cutoff scores [63, 67] we observed 8.1% of pregnant persons had mild or moderate depressive symptoms (EPDS *≥* 11) [63], 5.7% of pregnant persons had mild or moderate state anxiety symptoms (STAI state scale *≥* 40) [67], and 13.8% of pregnant persons had mild or moderate trait anxiety symptoms (STAI trait scale *≥* 40) [67]. Infants were predominately female (54%) and born at term age (*≥* 37 weeks’ gestation) (93.5%). Finally, infant omega-3 levels were normally distributed (Fig. 1).

**Table 1:** Participant demographic information (*N* = 384)

**Maternal Characteristics**	
**Maternal Race**	
American Indian or Alaskan Native	50 (13.0%)
Black or African American	1 (0.3%)
Native Hawaiian or Other Pacific Islander	1 (0.3%)
White	326 (84.9%)
Other or Unknown	6 (1.6%)
**Maternal Ethnicity**	
Hispanic or Latino	18 (4.7%)
Non-Hispanic or Latino	366 (95.3%)
**Maternal Educational Attainment**	
Some Primary School	2 (0.5%)
Completed Primary School	31 (8.1%)
Some High School	47 (12.2%)
Completed High School	95 (24.7%)
Completed College	138 (35.9%)
Postgraduate Degree	71 (18.5%)
**Marital or Partner Status**	
Married or Partnered	357 (93.0%)
Not Married or Partnered	27 (7.0%)
**Maternal Age at Delivery**	
Mean (SD)	28.3 (4.67)
Median [Min, Max]	29.0 [16.0, 42.0]
**Parity**	
0	136 (35.4%)
1	119 (31.0%)
2	82 (21.4%)
3+	47 (12.3%)
**Median Monthly Household Income Per Person**	
Mean (SD)	1160 (650)
Median [Min, Max]	1170 [62.5, 2500]
Missing	4 (1.0%)
**Healthcare Coverage**	
Commercial Health Insurance/Commercial HMO	260 (67.7%)
Public Assistance	119 (31.0%)
Self-pay	5 (1.3%)
**Pre-pregnancy overweight or obesity**	
Non-overweight or obese	173 (45.1%)
Overweight or obese	211 (54.9%)
**Prenatal Maternal EPDS**	
Mean (SD)	4.70 (3.83)
Median [Min, Max]	4.00 [0, 19.0]
No or low depression (EPDS < 11)	353 (91.9%)
Mild or Moderate depression (EPDS *≥* 11)	31 (8.1%)
**Prenatal Maternal STAI State Anxiety**	
Mean (SD)	26.0 (6.88)
Median [Min, Max]	24.0 [20.0, 56.0]
No or low state anxiety (STAI state scale < 40)	359 (93.5%)
Mild or moderate state anxiety (STAI state scale *≥* 40)	22 (5.7%)
Missing	3 (0.8%)
**Prenatal Maternal Trait Anxiety**	
Mean (SD)	30.7 (8.15)
Median [Min, Max]	29.0 [20.0, 64.0]
No or low trait anxiety (STAI trait scale < 40)	326 (84.9%)
Mild or moderate trait anxiety (STAI trait scale *≥* 40)	53 (13.8%)
Missing	5 (1.3%)
**Infant Characteristics**	
**Infant Assigned Sex at Birth**	
Female	208 (54.2%)
Male	176 (45.8%)
**Infant Gestational Age Category**	
Term-age (*≥* 37 weeks’ gestation)	359 (93.5%)
Preterm (< 37 weeks’ gestation)	25 (6.5%)
**Infant Gestational Age at Birth**	
Mean (SD)	39.2 (1.40)
Median [Min, Max]	39.3 [32.4, 42.4]

#### Association of pre-pregnancy BMI category and prenatal maternal depressive symptoms with the infant cord blood omega-3 index

**Fig. 2:**
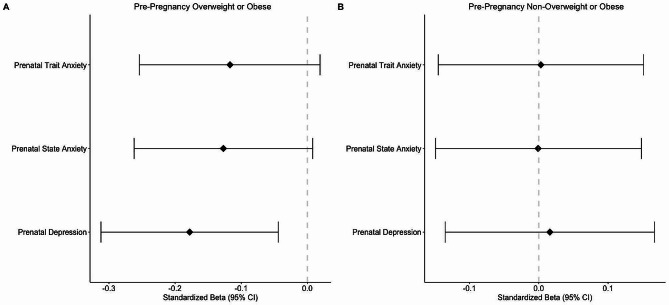
Association of Prenatal Maternal Depression and Anxiety Stratified by Pre-Pregnancy Body Mass Index Category. Panel A is stratified by pre-pregnancy overweight or obesity and Panel B is stratified by pre-pregnancy non-overweight or obesityThe y-axis depicts prenatal trait anxiety, prenatal state anxiety, and prenatal depressive symptoms. The x-axis depicts the standardized beta coefficient with 95% confidence intervals.

Linear regression models showed an interaction effect of pre-pregnancy BMI categories and prenatal maternal depressive symptoms on the infant cord blood omega-3 index in the unadjusted model (Interaction β = -0.07 ± 0.03, *F* (3, 380) = 5.42, *p <* .005, adj. R^2^ = 0.03) and adjusted model (Interaction β = -0.07 ± 0.03, *F* (5, 378) = 4.96, *p <* .001, adj. R^2^ = 0.05). Among pregnant people with pre-pregnancy OWOB, increased prenatal maternal depressive symptoms were associated with decreased infant cord blood omega-3 levels (unadjusted β = -0.06 ± 0.02; adjusted β = -0.07 ± 0.02) (Fig. 2). Preterm birth, but not infant assigned sex at birth, was a significant predictor in the adjusted interaction model. Preterm birth was associated with lower infant cord blood omega-3 levels (β = -0.74 ± 0.26).

#### Association of pre-pregnancy BMI category and prenatal maternal state anxiety symptoms with the infant cord blood omega-3 index

We did not observe an interaction effect of pre-pregnancy OWOB and prenatal maternal state anxiety symptoms on the infant cord blood omega-3 index in unadjusted or adjusted models. Main effects of pre-pregnancy BMI category were observed in the unadjusted (*F* (2, 378) = 5.02, *p <* .01, adj. R^2^ = 0.02) and adjusted models (*F* (4, 376) = 4.38, *p <* .01, adj. R^2^ = 0.03). Pre-pregnancy OWOB was associated with decreased infant cord blood omega-3 levels (unadjusted β = -0.34 ± 0.13; adjusted β = -0.35 ± 0.13). We did not observe a main effect of prenatal maternal state anxiety symptoms on infant cord blood omega-3 levels. Preterm birth was associated with lower infant cord blood omega-3 levels (β = -0.69 ± 0.26). Finally, infant assigned sex at birth was not a significant predictor in the adjusted model.

#### Association of pre-pregnancy BMI category and prenatal maternal trait anxiety symptoms with the infant cord blood omega-3 index

We did not observe an interaction effect of pre-pregnancy OWOB and prenatal maternal trait anxiety symptoms on the infant cord blood omega-3 index in unadjusted or adjusted models. Main effects of pre-pregnancy BMI category were observed in the unadjusted (*F* (2, 376) = 5.02, *p <* .01, adj. R^2^ = 0.02) and adjusted models (*F* (4, 374) = 4.38, *p <* .01, adj. R^2^ = 0.03). Pre-pregnancy OWOB was associated with decreased infant cord blood omega-3 levels (unadjusted β = -0.35 ± 0.13; adjusted β = -0.36 ± 0.13). We did not observe a main effect of prenatal maternal trait anxiety symptoms on infant cord blood omega-3 levels. Preterm birth was associated with lower infant cord blood omega-3 levels (β = -0.70 ± 0.26). Finally, infant assigned sex at birth was not a significant predictor in the adjusted model.

## Discussion

To summarize our findings examining the interactive effects of pre-pregnancy overweight or obesity and prenatal maternal mental health symptoms on infant cord blood omega-3 levels, we observed a significant interaction between pre-pregnancy body mass index and prenatal maternal depressive symptoms with cord blood omega-3. Our stratified models revealed that there was an association between prenatal maternal depressive symptoms with lower cord blood omega-3 levels only among individuals with pre-pregnancy overweight or obesity. We did not observe an interaction effect between pre-pregnancy overweight or obesity and prenatal maternal state or trait anxiety on infant cord blood omega-3 levels. Additionally, we did not observe a main effect of prenatal maternal state or trait anxiety on infant cord blood omega-3 levels. Prior research has demonstrated pre-pregnancy obesity is associated with higher intake of LC-PUFA, but a weaker association between dietary intake and respective maternal and infant cord blood LC-PUFA levels [69] whereas other studies have implicated lower prenatal maternal omega-3 intake and plasma levels as a risk factor for perinatal maternal depression [45–48]. Our findings, along with prior research in this domain suggest that pre-pregnancy obesity may impact the metabolism of FAs during pregnancy, therefore impacting the quantity of FAs neonates receive via the placental transfer in the womb.

Within our current context, low omega-3 in the fetal and neonatal periods have important implications for further development across the lifespan. It is well known that DHAs are an important structural component of the human brain. DHA plays important roles in cell signaling, the regulation of gene expression, and neurotransmission [70–72]. In humans, approximately 20% of the dry weight of the brain is made up of polyunsaturated fatty acids and one out of every three fatty acids in the nervous system is a polyunsaturated fatty acid (50% of these being DHAs) [73]. A recent study in humans showed an early association of LCPUFA-related genotypes with cognitive performance at school age after correction for current DHA blood concentrations, demonstrating possible programming effects of these nutrients on the brain development [74]. Recent studies have also found significant associations between higher concentrations of cord blood omega-3 fatty acids with longer birth length and infant growth [28], better hand-eye coordination [75], and lower scores on a parent-completed behavioral screen at age 10 [76].

In light of these findings, our study’s observation of the associations between pre-pregnancy obesity, prenatal maternal mental health symptoms, and infant cord blood omega-3 levels adds a crucial dimension to the existing literature. Specifically, our results highlight the compounded risk factors that can adversely affect fetal omega-3 status and subsequent developmental outcomes. Omega-3 deprivation has been further explored in animal models. It has been demonstrated in rodents that decreased plasma concentrations of DHA during development alter neurobiological pathways, which has long-term negative consequences on behavior [77]. Additionally, low levels of omega-3s in plasma led to altered physiology of neurotransmitters such as serotonin and dopamine [78], as well as decreased neuron size in various areas of the brain such as the hypothalamus and the hippocampus [79]. In additional animal models, omega-3 fatty acid deficiency has been found to lead to low levels of DHA in the cerebral cortex of the offspring and affect learning ability [80, 81]. These varying impacts in individual areas of function in the brain emphasize the importance of ensuring quantity of FA absorption by neonates during pregnancy. In light of our main finding, it is vital for clinicians overseeing the healthcare of pregnant individuals who are overweight, obese, and/or susceptible to prenatal depressive symptoms to counsel them on the critical significance of sufficient maternal dietary intake of omega-3s.

To our knowledge, the present analysis is the first to date to examine associations between pre-pregnancy obesity, prenatal maternal mental health symptoms, and infant cord blood omega-3 levels. Due to the widespread prevalence of overweight and obesity before pregnancy worldwide, the detrimental effects for both mother and offspring constitute a significant public health concern. In the present analysis, the prevalence of pre-pregnancy obesity was 28.2%, mirroring the reported averages of the United States as a whole (30.0%) and of South Dakota specifically (28.0%) [82]. A total of 25.4% of our sample had a BMI classified as overweight prior to pregnancy which aligns with both the estimated national average (25.8%) and South Dakota’s estimated average (25.7%) [83]. As found in the present study, pre-pregnancy BMI negatively affects several fetal LC-PUFA profiles [84, 85]. These findings are consistent with previous research indicating that maternal obesity can influence fetal fatty acid composition, potentially impacting infant development. Longitudinal studies have shown that children born to mothers with obesity have increased risk of cognitive impairments [22]. Compared with children of mothers with BMI classified as normal weight, children of mothers classified as OWOB prior to pregnancy are at increased risk for compromised neurodevelopmental outcomes such as ADHD, autism spectrum disorder, developmental delay, and/or emotional/behavioral problems [12, 86, 87]. The magnitude of the overall effect of pre-pregnancy OWOB on child neurodevelopment is largely unknown, which places a continued emphasis on elucidating the mechanisms by which these associations affect subsequent childhood outcomes.

### Strengths and limitations

Significant strengths of our analysis include our moderate sample size and underrepresented research sample of pregnant persons from a rural part of the United States, including a portion who identify as American Indian/Alaskan Indian. However, several limitations and potential sources of bias may have influenced our findings. In the PASS study, the collected cord blood comprised a combination of arterial and venous blood, and it is possible that omega-3 levels may vary depending on the type of cord blood collected. In the current study, we did not have information regarding prenatal maternal diet to examine associations between prenatal maternal omega dietary intake with infant cord blood omega-3 levels. We additionally did not collect prenatal maternal blood samples which resulted in the inability to examine associations between prenatal maternal diet, maternal plasma omega-3 levels, and infant cord blood omega-3 levels. Inclusion in the present analysis required non-missing data on prenatal maternal depressive and anxiety questionnaires and the collection of infant cord blood. It is possible that individuals with complete data do not accurately reflect PASS at large. Further, it is possible that our findings, which draw from a predominately White and American Indian/Alaskan Indian rural sample of pregnant persons, will not generalize to other populations. Additionally, levels of maternal prenatal depressive and anxiety symptoms were low in this sample. Finally, there are several factors which should be explored in future analyses such as the association of infant cord blood omega-3 levels with subsequent infant or childhood health or neurobehavioral outcomes. There may be also factors that confound these results such as socioeconomic status, access to healthy foods, and access to prenatal care.

## Conclusions

Despite these limitations, our findings have important clinical and research implications: They underscore the importance of identifying potential pathways by which maternal metabolic and mental health impacts infant development. Additionally, due to the joint association of pre-pregnancy OWOB and perinatal depressive symptoms on infant cord blood omega-3 levels, future studies should continue examine this relation longitudinally to understand the potential mediating role of infant cord blood omega-3 levels on child health or neurodevelopmental outcomes. Further understanding of such mechanisms may inform intervention research to elucidate causal associations between prenatal maternal omega-3 intake among individuals with metabolic or mental health conditions during pregnancy on adverse birth, infant, or child developmental outcomes.

## Data Availability

Select de-identified data from the ECHO Program are available through NICHD’s Data and Specimen Hub (DASH) [https://dash.nichd.nih.gov/]. Information on study data not available on DASH, such as some Indigenous datasets, can be found on the ECHO study DASH webpage [https://dash.nichd.nih.gov/explore/study?q=echo&filters=%5b%5d&page=1&sortBy=relevance&asc=true&size=50].

## Abbreviations

OWOB: Overweight or obesity
non-OWOB: Not overweight or obese
LC-PUFA: Long-chain Polyunsaturated Fatty Acid
PUFA: Polyunsaturated Fatty Acid
FA: Fatty acid
EPA: Eicosapentaenoic acid
DHA: Docosahexaenoic acid
BMI: Body Mass Index
EPDS: Edinburgh Postnatal Depression Scale
STAI: State-Trait Anxiety Inventory
